# Longitudinal associations between psychedelic use and unusual visual experiences in the United States and the United Kingdom

**DOI:** 10.1177/02698811231218931

**Published:** 2023-12-23

**Authors:** Otto Simonsson, Peter S Hendricks, Cecilia UD Stenfors, Simon B Goldberg, Ludwig Honk, Walter Osika

**Affiliations:** 1 Department of Neurobiology, Care Sciences, and Society, Karolinska Institutet, Stockholm, Sweden; 2 Department of Sociology, University of Oxford, Oxford, UK; 3 Department of Health Behavior, School of Public Health, University of Alabama at Birmingham, Birmingham, AL, USA; 4 Department of Psychology, Stockholm University, Stockholm, Sweden; 5 Department of Counseling Psychology, University of Wisconsin—Madison, Madison, WI, USA

**Keywords:** Psychedelics, psilocybin, LSD, mescaline, risks, visual, HPPD

## Abstract

**Background:**

Whereas findings from case reports and cross-sectional studies suggest that naturalistic psychedelic use may be associated with unusual visual experiences that occur after the acute pharmacological effects have subsided, such findings need to be replicated in longitudinal studies to better understand potential cause-and-effect relationships.

**Aims:**

To investigate longitudinal associations between naturalistic psychedelic use and unusual visual experiences.

**Methods:**

Using a longitudinal observational research design with samples representative of the US and UK adult populations with regard to sex, age, and ethnicity (*N* = 9732), we investigated the relationship between psychedelic use during the 2-month study period and changes in past-week unusual visual experiences.

**Results:**

The follow-up survey was completed by 79% of respondents (*n* = 7667), with 100 respondents reporting psychedelic use during the 2-month study period (1.3% of those who responded at follow-up). In covariate-adjusted regression models, the results showed that, as hypothesized, psychedelic use during the 2-month study period was associated with greater increases in unusual visual experiences. Notably, there was an interaction between lifetime psychedelic use and psychedelic use during the study period on unusual visual experiences such that those who used psychedelics for the first time reported greater increases in unusual visual experiences.

**Conclusions:**

Psychedelic use may elicit unusual visual experiences that occur after the acute pharmacological effects have subsided, especially among those who have not used psychedelics previously. Future longitudinal studies are warranted to further elucidate these relationships.

Psychedelics such as psilocybin and lysergic acid diethylamide (LSD) have shown promise as potential treatments for various psychiatric disorders (Nutt and Carhart-Harris, 2021). For example, in a recent randomized controlled trial, psilocybin with psychotherapy produced significant decreases in the percentage of heavy drinking days, compared with an active placebo medication (diphenhydramine) with psychotherapy, in patients with alcohol use disorder (Bogenschutz et al., 2022). The evidence to date suggests that these substances have a favorable risk profile when administered in clinical settings (Holze et al., 2021; Roscoe and Lozy, 2022; Simonsson et al., 2023), but relatively little remains known about the risk-benefit ratio of psychedelic use outside of the tightly controlled settings of modern-day clinical trials. It is therefore important to investigate potential risks associated with naturalistic psychedelic use, especially given that psychedelic use in the general population has increased in recent years (Livne et al., 2022).

While the whole range of risks associated with psychedelic use remains to be fully understood (Schlag et al., 2022), one common concern is that psychedelics may cause visual hallucinations or flashback-type phenomena that occur after the pharmacological effects have subsided (e.g., halos around objects, macropsia, micropsia; Halpern and Pope, 2003). These types of unusual visual experiences can meet the DSM-IV-TR criteria for hallucinogen persisting perception disorder (HPPD) if they persist and cause significant distress or impairment in daily functioning and other medical or psychiatric conditions can be ruled out (American Psychiatric Association, 2013; see Halpern et al., 2018 for proposed HPPD subtypes). The prevalence estimates of HPPD in the general population varies (Baggott et al., 2011; Halpern et al., 2018; see also Vis et al., 2021), but in a recent cross-sectional study with a sample representative of the US adult population with regards to sex, age, and ethnicity, 1.3% of respondents who had used psychedelics in the past reported having been told by a doctor or medical professional that they had HPPD (Simonsson et al., 2023).

The cardinal diagnostic criteria for HPPD includes the presence of visual hallucinations or flashback-type phenomena, highlighting the need to investigate if, for whom, and under what circumstances naturalistic psychedelic use might lead to unusual visual experiences, even if it is not associated with impairment or distress. The prevalence of visual hallucinations or flashback-type phenomena has been examined in controlled psychedelic studies with healthy volunteers (Müller et al., 2022), but the studies on naturalistic psychedelic use and unusual visual experiences have mostly relied on case reports and cross-sectional research designs (Baggott et al., 2011; Halpern et al., 2018; Krebs and Johansen, 2013), which limits inferences about possible causal relationships. It is therefore important to use longitudinal research designs with large and ideally nationally representative samples to investigate potential cause-and-effect relationships between naturalistic psychedelic use and unusual visual experiences.

Using a longitudinal observational research design with samples representative of the US and UK adult population with regard to sex, age, and ethnicity (*N* = 9732), we investigated the relationship between psychedelic use and unusual visual experiences. We hypothesized that respondents who reported psychedelic use during the 2-month study period would have a greater increase in unusual visual experiences than respondents who did not report psychedelic use in the same time period.

## Materials and methods

### Participants and procedure

The study (hypotheses, design plan, sampling plan, variables, and analysis plan) was preregistered on the Open Science Framework at https://osf.io/czmfy. Exploratory analyses were not preregistered. The participants (18 years or older) were recruited on Prolific Academic (https://app.prolific.co) and were US (*N* = 4867) and UK (*N* = 4865) residents. The study description in recruitment materials did not mention psychedelic use (see Supplemental Materials for recruitment materials) to avoid self-selection bias. The representativeness function on Prolific Academic was used to stratify the samples across sex (Male, Female), age (18–27, 28–37, 38–47, 48–57, 58+), and ethnicity (White, Mixed, Asian, Black, Other) to reflect the demographic distribution of the US and UK adult populations. In August 2022, respondents were asked to complete the baseline survey (T1), which included items related to demographic characteristics, psychedelic use, and unusual visual experiences (see Supplemental Materials for survey items used in this study). Approximately 2 months later (October 2022), respondents were invited to complete the follow-up survey (T2), which included items related to psychedelic use, use of other substances, and unusual visual experiences. This study was part of a larger survey and completion of the baseline survey resulted in £0.9 payment and completion of the follow-up survey resulted in £0.9 payment. Study procedures were determined to be exempt from review by the Institutional Review Board at the University of Wisconsin—Madison.

### Measures

#### Demographics

At T1, all respondents were asked to report age, gender identity, educational attainment, religiosity, and political affiliation.

#### Psychedelic use

At T1, all respondents were asked to report which, if any, of the following psychedelics they had ever used: ayahuasca, N,N-Dimethyltryptamine, psilocybin, LSD, mescaline, peyote, or San Pedro. Those who reported lifetime psychedelic use were also asked to report which, if any, of these substances that they had used in the past 2 months. At T2, all respondents were asked which, if any, of these same substances that they had used in the past 2 months (i.e., in the time between T1 and T2).

#### Use of other substances

At T2, all respondents were asked to report which, if any, of the following substances they had used in the past 2 months (i.e., in the time between T1 and T2): alcohol, nicotine products (e.g., cigarettes, e-cigarettes, cigarillos, little cigars, smokeless tobacco), cannabis products (e.g., “weed,” THC, CBD, hemp oil), MDMA, major stimulants (e.g., cocaine, methamphetamine), illicit narcotic analgesics/opioids (e.g., morphine, heroin, oxycodone), illicit benzodiazepines and barbiturates (e.g., Valium, Alprazolam (Xanax)), inhalants (poppers, whip-its, nitrous oxide, glue), and other substances.

#### Unusual visual experiences

At T1 and T2, all respondents completed a 9-item questionnaire used by Baggott et al. (2011) that includes items related to unusual visual experiences (e.g., “Halos or auras around things,” “Things that are moving leave afterimages behind”), excluding times when the respondents had ingested strong psychoactive substances within the past 3 days or the respondents were in a trance, falling asleep, waking up, or had not slept. The original questionnaire was modified to only include unusual visual experiences in the past 7 days, which was done to increase the likelihood that psychedelic use during the 2-month study period happened prior to the period they were reporting on their unusual visual experiences (i.e., that psychedelic use preceded any potential change in unusual visual experiences). The response options for each item were dichotomous (yes, no) and the total score was calculated by summing across items. Higher scores indicate a greater number of unusual visual experiences. The internal consistency was adequate (Cronbach’s alpha = 0.74 and 0.78 at T1 and T2, respectively).

As an additional item, all respondents were also asked at T1 and T2 about sightings in the past 7 days of unidentified aerial phenomena (UAP; i.e., observations of events in the sky that cannot be identified as aircraft or known natural phenomena), which has recently become the object of scientific investigation (Watters et al., 2023). The response options for the item were dichotomous (yes, no). While several UAP sightings have reportedly involved visual observations by military personnel and corroborative observations via multiple sensors (Watters et al., 2023), it is plausible that many UAP sightings can be explained as unusual visual experiences (e.g., visual hallucinations), which was the rationale for including this additional item. There was a moderate point-biserial correlation between the UAP variable and the total score for unusual visual experiences (*r_pb_* = 0.27 and 0.28 at T1 and T2, respectively, *ps* < 0.001).

#### Statistical analyses

As specified in the preregistration, we used multiple linear regression to assess whether there were significant differences in past-week unusual visual experiences change scores (i.e., from T1 to T2) between those who reported psychedelic use during the 2-month study period versus those who did not, controlling for age (recoded as: 18–27, 28–37, 38–47, 48–57, 58+), gender (recoded as: male, female, other), educational attainment (no Bachelor’s degree, Bachelor’s degree, or higher), degree of religiosity (not at all religious, a little religious, moderately religious, quite religious, very religious), political affiliation (Democratic Party or Republican Party (for US respondents); Remain side or leave side (for UK respondents)), past 2-month use of alcohol, nicotine products, cannabis products, MDMA, major stimulants, illicit narcotic analgesics/opioids, illicit benzodiazepines and barbiturates, inhalants, and other substances at T2 (each substance entered as a separate covariate), and psychedelic use in the past 2 months at T1. These control variables were preregistered and chosen based on a previous longitudinal study on psychedelic use (Forstmann et al., 2020). As an exploratory analysis, we used multiple linear regression to test whether there was an interaction between psychedelic use during the study period and lifetime psychedelic use prior to the study on unusual visual experiences. Sensitivity analyses were conducted using zero-inflated models.

In addition to the analyses on unusual visual experiences, we also conducted exploratory analyses on past 7 days UAP sightings using multiple logistic regression (with the same control variables as above). First, among those who reported no past 7 days UAP sightings at T1, we assessed whether there were significant differences in past 7 days UAP sightings at T2 between those who reported psychedelic use during the study period versus those who did not. Second, among those who reported no past 7 days UAP sightings at T1, we tested whether there was an interaction between psychedelic use during the study period and lifetime psychedelic use prior to the study on past 7 days UAP sightings at T2.

For all analyses, *p*-values are reported with three decimal places, allowing the reader to estimate any *p*-value corrections of the reader’s choosing. At T1, there were no missing data. At T2, missing data was addressed by using Multivariate Imputation by Chained Equations (MICE; Van Buuren & Groothuis-Oudshoorn, 2011). The MICE package version 3.15.0 in R Studio (https://cran.r-project.org/web/packages/mice/index.html) was used to impute the missing data 20 times using random forest imputations as method. We subsequently replaced imputed values on hierarchical variables (i.e., variables that should have missing data by design) before we analyzed the data (using functions with() and pool()). Models were run across imputations and pooled according to Rubins’ (1976) rules using the “pool” function in the “mice” package.

## Results

Of the 9732 respondents who completed the survey at T1, 7667 respondents completed the survey at T2 (79% retention rate) and 100 respondents reported psychedelic use during the 2-month study period (1.3% of those who completed the survey at T2).

Table 1 shows sample characteristics at baseline. As shown in the table, among those who reported psychedelic use during the study period, 81% reported having used psychedelics prior to the study, which was significantly higher than those who did not report psychedelic use during the study period (17%). Notably, most of the items related to past-week unusual visual experiences (including UAP sightings) were significantly more common among respondents who reported psychedelic use during the study period than those who did not (see Supplemental Tables 1 and 2 for descriptive statistics of past-week unusual visual experiences and UAP sightings).

**Table 1.** Sample characteristics of non-users (*n* = 9632) and users (*n* = 100).

Variables	Non-users (%)	Users (%)	*p*
Age			
18–27	20.5	24.0	<0.001
28–37	22.1	35.0	
38–47	18.5	24.0	
48–57	15.7	15.0	
58+	23.2	2.0	
Gender identity			
Male	47.6	63.0	<0.001
Female	50.9	33.0	
Other	1.5	4.0	
Educational attainment			
Bachelor’s degree or higher	56.8	63.0	0.209
Less than bachelor’s degree	43.3	37.0	
Religiosity			
Not at all religious	55.8	75.0	0.004
A little religious	19.0	13.0	
Quite religious	11.7	6.0	
Moderately religious	8.6	4.0	
Very religious	5.0	2.0	
Political affiliation			
Democratic party	34.7	59.0	<0.001
Republican party	15.1	17.0	
Remain side	34.6	21.0	
Leave side	15.7	3.0	
Lifetime substance use			
Psychedelics	17.1	81.0	<0.001
Alcohol	82.4	91.0	0.025
Nicotine products	51.3	81.0	<0.001
Cannabis products	53.1	87.0	<0.001
MDMA	12.4	63.0	<0.001
Major stimulants	16.7	59.0	<0.001
Illicit narcotic analgesics or opioids	7.5	35.0	<0.001
Illicit benzodiazepines and barbiturates	10.0	52.0	<0.001
Inhalants	9.7	43.0	<0.001
Other substances	3.7	26.0	<0.001
Past-week unusual visual experiences			
Halos or auras around things	2.4	6.0	0.019
Stationary things appear to move, breathe, grow, or shrink	3.1	5.0	0.282
Things that are moving appear to be not moving	2.0	4.0	0.168
Things that are moving leave afterimages behind	2.3	7.0	0.002
Colors increase in brightness or intensity	4.4	13.0	<0.001
You see with open eyes patterns or textures that are not really there	3.7	9.0	0.006
You see with open eyes things or objects that are not really there	2.0	2.0	0.998
Oscillations or flashing light sources, as in TVs or fluorescent lights, bother you more than other times in your life	6.6	14.0	0.003
Grids, gratings or closely spaced lines bother you more than other times in your life	2.1	7.0	0.001
Unidentified aerial phenomena (i.e., observations of events in the sky that cannot be identified as aircraft or known natural phenomena)	1.0	3.0	0.049

This table shows sample characteristics at baseline of respondents who did not report psychedelic use during the study period (i.e., non-users) and respondents who did (i.e., users). Pearson’s Chi-squared tests were used to examine the characteristics of users versus non-users. All percentages were rounded to the nearest 0.1%; cumulative percentages may not add to 100.0.

Table 2 displays results from the multiple regression models testing the association between psychedelic use during the 2-month study period and unusual visual experiences change scores (see Supplemental Table 3 for results on UAP sightings). As indicated in the table, psychedelic use during the study period was associated with greater increases in unusual visual experiences. Notably, there was an interaction between lifetime psychedelic use and psychedelic use during the study period on unusual visual experiences such that those who used psychedelics for the first time reported greater increases in unusual visual experiences (see Supplemental Table 4 for adjusted means). Sensitivity analyses produced similar results.

**Table 2.** Multiple imputation regression model estimates—past-week unusual visual experiences.

Models	B (CI 95%)	*p*
Psychedelic use during study period	0.33 (0.15–0.52)	<0.001
Psychedelic use during study period × lifetime psychedelic use	−1.08 (−1.50 to −0.65)	<0.001

B: unstandardized beta; the linear regression models controlled for age, gender, educational attainment, degree of religiosity, political affiliation, past 2 months use of alcohol, nicotine products, cannabis products, MDMA: major stimulants, illicit narcotic analgesics/opioids, illicit benzodiazepines and barbiturates, inhalants, and other substances at T2, and psychedelic use in the past 2 months at T1. See Supplemental Table 5 for results on unimputed data.

## Discussion

This longitudinal study investigated the relationship between naturalistic psychedelic use and unusual visual experiences in samples representative of the US and UK adult populations with regard to sex, age, and ethnicity. The results showed, as hypothesized, that psychedelic use during the 2-month study period was associated with greater increases in unusual visual experiences, which broadly corresponds with previous findings (Baggott et al., 2011; Halpern and Pope, 2003; but see Krebs and Johansen, 2013). Notably, there was an interaction between lifetime psychedelic use and psychedelic use during the study period on unusual visual experiences such that those who used psychedelics for the first time reported greater increases in unusual visual experiences. While prior research has investigated associations between frequency of past psychedelic use and unusual visual experiences (e.g., Baggott et al., 2011; Stanton and Bardoni, 1972), these results contribute to a limited research literature on unusual visual experiences following first time psychedelic use. Taken together, these findings indicate that naturalistic psychedelic use may increase unusual visual experiences that occur after the acute pharmacological effects have subsided, especially among those who have not used psychedelics previously.

The current results suggest that public health campaigns communicate to those who intend on using psychedelics (e.g., in states that have liberalized laws around psilocybin and other psychedelic substances) that unusual visual experiences are possible consequences of use. While such experiences may not necessarily be associated with impairment or distress, clinicians should be prepared for reports of unusual visual experiences among patients who report recent psychedelic use and monitor these symptoms over time so as to determine the best course of action. Future longitudinal research should investigate the time course of these unusual visual experiences and if, for whom, and under what circumstances they might represent a significant adverse outcome requiring intervention.

There are several limitations to consider when interpreting the results. First, no conclusive causal inferences can be made due to the observational study design. Second, the recruited sample was stratified across sex, age, and ethnicity to reflect the US and UK adult populations, but it might not have been representative of other variables such as income or educational attainment. Third, the covariate-adjusted regression models in this study controlled for only a subset of potential confounders. It is still possible that unmeasured confounding variables (e.g., prescribed medication use during the study period) could have influenced the results. Fourth, the retention rate at T2 was 79%. It is therefore possible that the results were influenced by attrition bias. Although we used multiple imputations, which is robust to data missing at random (i.e., missingness conditional on observed variables), it is possible that data were missing not at random (i.e., nonignorable missingness that is not recaptured with observed values; Graham, 2009). Fifth, all variables were measured using self-report and respondents were asked to retrospectively report psychedelic use and unusual visual experiences, which may have biased responses. Sixth, the questionnaire used to measure unusual visual experiences has been used in previous psychedelic research, which allows for comparisons between studies, but future studies would benefit from also using a more thoroughly standardized unusual visual experiences questionnaire.

## Conclusions

This study used a longitudinal observational research design to build on previous studies that have cross-sectionally investigated the association between naturalistic psychedelic use and unusual visual experiences. In conclusion, the results in this study suggest that psychedelic use may elicit unusual visual experiences that occur after the acute pharmacological effects have subsided, especially among those who have not used psychedelics previously. Future longitudinal studies are warranted to further elucidate these relationships.

## Supplemental Material

sj-docx-1-jop-10.1177_02698811231218931 – Supplemental material for Longitudinal associations between psychedelic use and unusual visual experiences in the United States and the United Kingdom Click here for additional data file. Supplemental material, sj-docx-1-jop-10.1177_02698811231218931 for Longitudinal associations between psychedelic use and unusual visual experiences in the United States and the United Kingdom by Otto Simonsson, Peter S Hendricks, Cecilia UD Stenfors, Simon B Goldberg, Ludwig Honk and Walter Osika in Journal of Psychopharmacology
