# Supplementary material for: Longitudinal associations between psychedelic use and unusual visual experiences in the United States and the United Kingdom
Source: J Psychopharmacol. 2023 Dec 23;38(1):110–5. doi: 10.1177/02698811231218931 (PMC10851627; doi:10.1177/02698811231218931)
Supplement: sj-docx-1-jop-10.1177_02698811231218931 – Supplemental material for Longitudinal associations between psychedelic use and unusual visual experiences in the United States and the United Kingdom [file sj-docx-1-jop-10.1177_02698811231218931.docx]

**Supplemental Materials**

Recruitment Materials

Baseline

The aim of this study is to better understand factors (e.g., substance use) that predict health behavior. You will be required to complete a set of surveys assessing your health behaviors along with demographic measures. You will be asked sensitive questions (e.g., about substance use). To have your submission accepted, you must correctly answer questions designed to check if you are paying attention. Anonymized data may be made available to other researchers. Approximately two months after completing this study, you will be invited to complete a second study. Please ONLY participate in this study if you think you will be able to complete the second study.

Follow-Up

Thank you for completing our initial study!

In this follow-up study we are seeking to better understand factors (e.g., substance use) that predict health behavior. You are being invited because you completed our initial survey approximately 2-3 months ago. In this follow-up study, you will again be required to complete a set of surveys. You may be asked sensitive questions (e.g., about substance use). To have your submission accepted, you must also correctly answer questions designed to check if you are paying attention. Anonymized data may be made available to other researchers.

Survey Items

T1

What is your age?

- Slider scale (18-100)

What is your gender?

- Male
- Female
- Transgender (male to female)
- Transgender (female to male)
- Non-binary gender
- Other

Do you have a bachelor's degree or higher?

- Yes
- No

For UK respondents:

If you had to choose, do you think of yourself as closer to the Remain or Leave side in the EU referendum debate?

- Remain
- Leave

For US respondents:

If you had to choose, do you think of yourself as closer to the Democratic Party or the Republican Party?

- The Democratic Party
- The Republican Party

How religious are you?

- Not at all religious
- A little religious
- Moderately religious
- Quite religious
- Very religious

In your life, which of the following drugs have you EVER used? (select all that apply)

- Ayahuasca
- DMT
- LSD, also called ‘acid’
- Mescaline
- Peyote
- Psilocybin, also called ‘magic mushrooms’
- San Pedro
- Alcohol
- Nicotine products (e.g., cigarettes, e-cigarettes, cigarillos, little cigars, smokeless tobacco)
- Cannabis products (e.g., weed, THC, CBD, hemp oil)
- MDMA, also called 'ecstasy'
- Major stimulants (e.g., cocaine, methamphetamine)
- Illicit narcotic analgesics/opioids (e.g., morphine, heroin, oxycodone)
- Illicit benzodiazepines and barbiturates (e.g., Valium, Alprazolam [Xanax])
- Inhalants (poppers, whip-its, nitrous oxide, glue)
- Other substances
- None of the above

IF psychedelic use reported in previous question:

Over the past two months, which of the following drugs have you used? (select all that apply)

- Ayahuasca
- DMT
- LSD, also called ‘acid’
- Mescaline
- Peyote
- Psilocybin, also called ‘magic mushrooms’
- San Pedro

T2

Over the past two months, which of the following drugs have you used? (select all that apply)

- Ayahuasca
- DMT
- LSD, also called ‘acid’
- Mescaline
- Peyote
- Psilocybin, also called ‘magic mushrooms’
- San Pedro
- Alcohol
- Nicotine products (e.g., cigarettes, e-cigarettes, cigarillos, little cigars, smokeless tobacco)
- Cannabis products (e.g., weed, THC, CBD, hemp oil)
- MDMA, also called 'ecstasy'
- Major stimulants (e.g., cocaine, methamphetamine)
- Illicit narcotic analgesics/opioids (e.g., morphine, heroin, oxycodone)
- Illicit benzodiazepines and barbiturates (e.g., Valium, Alprazolam [Xanax])
- Inhalants (poppers, whip-its, nitrous oxide, glue)
- Other substances
- None of the above

T1 and T2

Not counting times when (1) you were inebriated or under the influence of any strong psychoactive; or (2) you had taken any of these substances within the last 3 days; or (3) you were in a trance, falling asleep, waking up, or had not slept in a long time, have you had a period in the past 7 days when you experienced any of the following visual effects/disturbances?

- Halos or auras around things
- Stationary things appear to move, breathe, grow, or shrink
- Things that are moving appear to be not moving
- Things that are moving leave afterimages behind
- Colors increase in brightness or intensity
- You see with open eyes patterns or textures that are not really there
- You see with open eyes things or objects that are not really there
- Oscillations or flashing light sources, as in TVs or fluorescent lights, bother you more than other times in your life
- Grids, gratings or closely spaced lines bother you more than other times in your life
- Unidentified aerial phenomena (i.e., observations of events in the sky that cannot be identified as aircraft or known natural phenomena)

| Supplemental Table 1. Descriptive statistics of past-week unusual visual experiences | | | | | |
| --- | --- | --- | --- | --- | --- |
|  | Unusual visual experiences (total score) | | | | |
|  | T1 | T2 | T1 to T2 change score | | |
|  | Mean (SD) | Mean (SD) | Mean (SD) | Skewness | Kurtosis |
| Full sample | 0.29 (0.91) | 0.29 (0.95) | 0.03 (0.88) | 1.00 | 29.99 |
| Non-users | 0.29 (0.90) | 0.28 (0.93) | 0.03 (0.86) | 0.86 | 30.62 |
| All psychedelic users | 0.67 (1.20) | 0.89 (1.69) | 0.22 (1.81) | 1.60 | 9.31 |
| Experienced psychedelic users | 0.72 (1.27) | 0.68 (1.31) | -0.04 (1.37) | -0.08 | 5.86 |
| First-time psychedelic users | 0.47 (0.84) | 1.79 (2.64) | 1.32 (2.83) | 1.42 | 4.08 |
| This table shows (unimputed) descriptive statistics of past-week unusual visual experiences. Note: Mean = the mean number of unusual visual experiences in the group; SD = Standard Deviation; Non-users = respondents who did not report psychedelic use during the study period; All psychedelic users = respondents who reported psychedelic use during the study period. Experienced psychedelic users = respondents who reported psychedelic use during the study period and who reported having tried psychedelics prior to the study. First-time psychedelic users = respondents who reported psychedelic use during the study period and who reported not having used psychedelics prior to the study. Note: T1 includes all respondents (N=9,732) while T2 and T1 to T2 change score includes only those respondents who also completed T2 (N=7,667). | | | | | |

| Supplemental Table 2. Descriptive statistics of past-week UAP sightings | | |
| --- | --- | --- |
|  | UAP sightings | |
|  | n (%) | |
|  | T1 | T2 |
| Full sample | 100 (1.0) | 78 (1.0) |
| Non-users | 97 (1.0) | 70 (0.9) |
| All psychedelic users | 3 (3.0) | 8 (8.0) |
| Experienced psychedelic users | 3 (3.7) | 3 (3.7) |
| First-time psychedelic users | 0 (0.0) | 5 (26.3) |
| This table shows (unimputed) descriptive statistics of past-week UAP sightings. Note: n = the number of respondents reporting UAP sightings in the group; % = the percentage of respondents reporting UAP sightings in the group. All percentages were rounded to the nearest 0.1%. Non-users = respondents who did not report psychedelic use during the study period; All psychedelic users = respondents who reported psychedelic use during the study period. Experienced psychedelic users = respondents who reported psychedelic use during the study period and who reported having tried psychedelics prior to the study. First-time psychedelic users = respondents who reported psychedelic use during the study period and who reported not having tried psychedelics prior to the study. Note: T1 includes all respondents (N=9,732) while T2 includes only those respondents who also completed T2 (N=7,667). | | |

| Supplemental Table 3. Regression model estimates – past-week UAP sightings | | | | |
| --- | --- | --- | --- | --- |
|  | Unimputed | | Imputed | |
|  | aOR (CI 95%) | *p* | aOR (CI 95%) | *p* |
| Psychedelic use during study period | 5.86 (1.71 – 17.1) | .002 | 6.93 (2.21 – 21.8) | <.001 |
| Psychedelic use during study period x lifetime psychedelic use | 0.04 (0.00 – 0.32) | .004 | 0.04 (0.00 – 0.35) | .004 |
| Note: aOR = adjusted odds ratio; the logistic regression models controlled for age, gender, educational attainment, degree of religiosity, political affiliation, past two month use of alcohol, nicotine products, cannabis products, MDMA, major stimulants, illicit narcotic analgesics/opioids, illicit benzodiazepines and barbiturates, inhalants, and other substances at T2, and psychedelic use in the past two months at T1. | | | | |

| Supplemental Table 4. Adjusted means from interaction model in Table 2 | | |
| --- | --- | --- |
|  | Previous psychedelic use | No previous psychedelic use |
|  | Adjusted mean change score (CI 95%) | |
| Psychedelic use during the study period | | |
| Yes | 0.08 (-0.14 – 0.30) | 1.21 (0.82 – 1.61) |
| No | -0.02 (-0.08 – 0.03) | 0.03 (0.01 – 0.06) |
| This table shows (unimputed) adjusted mean change score of past-week unusual visual experiences. | | |

| Supplemental Table 5. Regression model estimates – past-week unusual visual experiences (change scores) | | | | |
| --- | --- | --- | --- | --- |
|  | Unimputed | | Imputed | |
|  | B (CI 95%) | *p* | B (CI 95%) | *p* |
| Psychedelic use during study period | 0.33 (0.14 – 0.53) | <.001 | 0.33 (0.15 – 0.52) | <.001 |
| Psychedelic use during study period x lifetime psychedelic use | -1.08 (-1.53 – -0.63) | <.001 | -1.08 (-1.50 – -0.65) | <.001 |
| Note: B = unstandardized beta; the linear regression models controlled for age, gender, educational attainment, degree of religiosity, political affiliation, past two month use of alcohol, nicotine products, cannabis products, MDMA, major stimulants, illicit narcotic analgesics/opioids, illicit benzodiazepines and barbiturates, inhalants, and other substances at T2, and psychedelic use in the past two months at T1. | | | | |
